# Analysis of the *Prefoldin* Gene Family in 14 Plant Species

**DOI:** 10.3389/fpls.2016.00317

**Published:** 2016-03-15

**Authors:** Jun Cao

**Affiliations:** Institute of Life Science, Jiangsu UniversityZhenjiang, China

**Keywords:** prefoldin, phylogeny, evolution, functional divergence, expression

## Abstract

Prefoldin is a hexameric molecular chaperone complex present in all eukaryotes and archaea. The evolution of this gene family in plants is unknown. Here, I identified 140 *prefoldin* genes in 14 plant species. These prefoldin proteins were divided into nine groups through phylogenetic analysis. Highly conserved gene organization and motif distribution exist in each prefoldin group, implying their functional conservation. I also observed the segmental duplication of maize *prefoldin* gene family. Moreover, a few functional divergence sites were identified within each group pairs. Functional network analyses identified 78 co-expressed genes, and most of them were involved in carrying, binding and kinase activity. Divergent expression profiles of the maize *prefoldin* genes were further investigated in different tissues and development periods and under auxin and some abiotic stresses. I also found a few *cis*-elements responding to abiotic stress and phytohormone in the upstream sequences of the maize *prefoldin* genes. The results provided a foundation for exploring the characterization of the *prefoldin* genes in plants and will offer insights for additional functional studies.

## Introduction

Molecular chaperones can stabilize, interact with, or assist other proteins to acquire functionally active conformations, without being present in their final structures (Hartl, 1996). Chaperonins play a role in protein assembly, folding, trafficking and degradation, and are crucial for cellular development (Hartl et al., 2011). Group I chaperonins are found in eubacteria and endosymbiotic organelles, whereas group II ones are present in archaea and eukaryotes. They share a common structure with different functions (Yébenes et al., 2011).

Prefoldin is a group II chaperonin. Archaea prefoldin possesses two subunits (prefoldin α and β) and polymerizes to an α2β4 hexamer (Leroux et al., 1999). Eukaryotic prefoldin consists of six subunits; two α subunits (PFD3 and PFD5) and four β subunits (PFD1, PFD2, PFD4, and PFD6) (Siegert et al., 2000). The structure confers selective substrate specificity for target proteins. They form a jellyfish-like heterohexameric complex to deliver newly synthesized unfolded proteins to cytosolic chaperonins containing TCP-1 (CCT) for protein folding. In addition, prefoldin also protects unfolded proteins during CCT capturing and releasing proteins (Siegert et al., 2000; Möckli et al., 2007).

Previous studies have indicated that prefoldin plays a central role in cellular development. Through binding to tubulin and actin, yeast prefoldin facilitated productive folding and binding inside the chaperonin cavity. Deleting of single or multiple subunits of prefoldin in yeast usually results in disruption of cytoskeletal structures (Vainberg et al., 1998). Similarly, studies on prefoldin function have revealed that down regulation of prefoldin can decreased the level of endogenous α-tubulin by 95%, and that of actin by 30% (Lundin et al., 2008). Deletion of *prefoldin* genes results in cytoskeletal defects, slow growth and cold sensitivity in yeast (Geissler et al., 1998; Siegers et al., 1999). In *Caenorhabditis elegans*, reduction of functional prefoldins caused embryonic lethality, and silencing of prefoldin subunits 1, 2, 3, and 6 exhibited reduced microtubule growth (Lundin et al., 2008). Mutation of prefoldin 5 or prefoldin 1 leads to a variety of neurodegenerative effects through a reduction of microfilaments and microtubules, such as mucus clearance defects, hydrocephaly, and loss of nerve bundles, in mice (Cao et al., 2008; Lee et al., 2011). In *Arabidopsis*, lesions in prefoldin 6 resulted in impaired microtubule organization and dynamics, which was associated with reduced plant size, defects in cell division, and so on (Gu et al., 2008). In addition, prefoldin 3 and prefoldin 5 mutants also displayed important changes in microtubule organization and developmental patterns. Furthermore, both mutants were sensitive to salt, implying important role of cytoskeleton in plant tolerance to salt stress (Rodríguez-Milla and Salinas, 2009). DELLA proteins directly interact with the prefoldin complex in a gibberellin (GA)-dependent manner. When GA is present, the complex is localized to the cytoplasm. When GA is absent, it stays in the nucleus, and then affects microtubule orientation (Locascio et al., 2013). A recent study has shown that prefoldin can bind chromatin and plays an important role in gene transcription (Millán-Zambrano et al., 2013). Bud27, an ATP-independent prefoldin-like molecular chaperone, can also regulate the gene expression transcribed by the RNA pol II in yeast (Mirón-García et al., 2014).

Six prefoldin members [PFD1 (At2g07340). PFD2 (At3g22480), PFD3 (At5g49510), PFD4 (At1g08780), PFD5 (At5g23290), and PFD6 (At1g29990)] have been identified in the *Arabidopsis* genome (Hill and Hemmingsen, 2001). Phylogenetic analysis showed that these six genes were divided into different evolutionary branches, suggesting the functional divergence among them (Hill and Hemmingsen, 2001). Moreover, only several of them have been functionally identified (Gu et al., 2008; Rodríguez-Milla and Salinas, 2009). Although prefoldin can form jellyfish-shaped hexameric complexes consisting of two α-type and four β-type subunits (Siegert et al., 2000; Martín-Benito et al., 2002), smaller individual motifs and more divergent patterns are not recognized among each prefoldin proteins. The recent availability of genome sequences of some models plant species provides an opportunity to study the evolution of *prefoldin* gene family. In this study, I have identified the *prefoldin* gene family from 14 plant species, and each species comprises 6–24 genes. Considering their significant developmental and physiological role, it is of considerable interest to us to study the evolution of plant *prefoldin* genes. Here, I performed integrated analyses to unravel the evolutionary mechanisms of the plant prefoldin protein family especially for maize. It will provide a useful basis for further functional studies of this gene family.

## Materials and Methods

### Prefoldin Sequence Retrieval and Identification in 14 Plant Species

To identify potential *prefoldin* gene in 14 completely sequenced plant genomes, I first used six *Arabidopsis* prefoldin sequences previous identified (Hill and Hemmingsen, 2001) as queries to perform BLAST searches against the phytozome database^1^ with -1 expect (E) threshold. In addition, a keyword “prefoldin” was also used to perform searching in this study. The Pfam database (Punta et al., 2012) was used to confirm the encoded prefoldin based on the presence or not of the prefoldin subunit domain. Next, the ProtParam tool^2^ and the CELLO v2.5 server^3^ (Yu et al., 2004) were used to determine the physicochemical parameters and subcellular localization of the prefoldin proteins, respectively.

### Phylogenetic Analyses of the *Prefoldin* Gene Family

To further explore the evolutionary relationship of plant prefoldins, multiple sequence alignments and phylogenetic analysis of the prefoldin proteins were performed with MUSCLE 3.52 (Edgar, 2004) and MEGA v5 (Tamura et al., 2011), respectively. And neighbor-joining (NJ) method was used to perform phylogenetic analyses of the prefoldin proteins with bootstrap analysis of 1,000 replicates (Cao, 2012). Furthermore, maximum likelihood and PhyML methods were also used to construct additional trees for validating the result from NJ tree.

### Chromosomal Location, Genomic Duplication and Inference of Duplication Time

I used the annotation data of the *prefoldin* genes on MaizeSequence^4^ for chromosomal location. Paralogous regions of the genomes were first predicted with SyMAP v3.4 (Soderlund et al., 2011). If two genes coming from the paraloguous regions were also located at the terminal evolutionary branch in the phylogenetic tree, they were thought to be derived from common ancestral duplication. To calculate the duplication time of the *prefoldin* paralogs, pairwise alignment of the maize *prefoldin* gene pairs was performed using an embedded program ClustalW (codons) in MEGA v5 (Tamura et al., 2011). Next, the *K_a_* and *K_s_* values of paralogous genes were estimated using K-Estimator 6.0 program (Comeron, 1999). The *K_s_* value was used to calculate the duplication date (T = *K_s_*/2λ), assuming clock-like rates (λ) of 6.5 × 10^-9^ for maize (Gaut et al., 1996).

### Exon-Intron Structure and Conserved Motifs Analysis

The organizations of *prefoldin* genes were analyzed in these plant lineages by comparing their coding and genomic sequence information in the Phytozome^5^ and NCBI databases. In addition, MEME program^6^ (Bailey et al., 2006) was used to identify finer motifs in the candidate plant prefoldin protein sequences. Parameter of maximum number of motifs is 15.

### Functional Divergence and Gene Co-expression Analyses

In the process of protein evolution, some residues are highly conserved, while others are highly variable. To further investigate the divergence between different groups of prefoldin proteins, DIVERGE (version 2.0) (Gu, 1999, 2001) was used to analyze the type-I functional divergence. The functional divergence between two groups was measured as the coefficient of functional divergence (θ). When the coefficient equals 0; it means that the evolutionary rate of the duplicate genes at each site is entirely consistent. Vice versa, when the coefficient is greater than 0, the evolutionary rate of the duplicate genes at some critical amino-acid residues is different. The software will predict these sites responding for the functional divergence.

To further analysis the relation between maize *prefoldin* and other genes, I also used the Co-expression Browser (COB)^7^ (Schaefer et al., 2014) to explore their networks. The domestication maize genotype was selected for co-expression analysis of the *prefoldin* genes. The network was built using expression profiles from 8-day seedlings. Expression matrices were used to generate profile correlations for the functional networks by calculating the Pearson correlation coefficient between each pair of gene expression profiles in each instance (Schaefer et al., 2014).

### Microarray-Based Expression Analysis

Maize microarray data (GSE27004) (Sekhon et al., 2011) were used for the expression analysis of the *prefoldin* genes. Expression data were normalized and viewed in the Genesis (v 1.7.6) program (Sturn et al., 2002).

### Plant Treatment, RNA Isolation, Quantitative Real-Time PCR (QRT-PCR), and Promoter Sequence Analysis

Endosperm can provide the necessary nutrition for early germination and growth when maize is grown in water. In this study, 10-day-old maize seedlings after germination were used to test the expression profiles of *prefoldin* genes under IAA, low temperature, drought, and salt stresses. For low temperature treatment, the maize seedlings were placed at 4°C environment for 3 h. And the seedlings were dried for 3 h between folds of tissue paper at 23 ± 1°C for drought treatment (Xia et al., 2012). The maize seedlings were put into 10 μM IAA and 150 mM NaCl solutions for 24 h for auxin and salt stress treatments, respectively. Control (CK) seedlings were normally grown at 23 ± 1°C with a photoperiod of 14 h light and 10 h dark. Three biological replicates were performed for qRT-PCR analysis. Total RNA was extracted with the TRIzol total RNA extraction kit (Sangon). RNase free DNase-I was used to remove genomic DNA. Next, I used M-MLV (TakaRa) to perform reverse transcription, followed by quantitative assays of each diluted cDNA using an ABI 7500 sequence detection system. All 13 maize *prefoldin* genes were selected for qRT-PCR analysis, and their primers are listed in Supplementary Table S2. *Actin 1* (*GRMZM2G126010*) gene in maize was used as the endogenous control. And 2^-ΔΔCT^ method (Livak and Schmittgen, 2001) was used to calculate their relative expression level. *t*-Test was used to perform a significant analysis.

To define the transcription start site (TSS) of each *prefoldin* gene in maize, I first collected their expressed sequence tag information. The TSS positions of *prefoldin* genes were used as references to determine their upstream promoter sequences. In this study, 1,000-bp upstream promoter sequences were acquired for further analyses. In addition, some abiotic stress- and phytohormone-responsive elements were identified in the promoter regions of the maize *prefoldin* genes using PLACE^8^ (Higo et al., 1999).

## Results and Discussion

### Identification of *Prefoldin* Genes in 14 Plant Species

To identify the *prefoldin* genes in plants, I first used the amino-acid sequences of *Arabidopsis* prefoldin (Hill and Hemmingsen, 2001) to perform BLAST searches in the phytozome database^9^. In addition, a keyword “prefoldin” search was also performed. All the putative prefoldin protein sequences were subjected to the Pfam analysis (Punta et al., 2012) to verify the reliability of the results based on the presence or not of the prefoldin subunit domain. As a result, I identified 140 *prefoldin* genes from 14 plants. The number of *prefoldin* genes ranged from 6 to 24 in each species (**Table 1**). There are 24 *prefoldin* genes existing in the soybean genome, while the members in other species range from about six in alfalfa, nine in *Arabidopsis*, sorghum, grape and *Physcomitrella patens*, 10 in rice, and 13 in maize and poplar. Previous study has identified 6 *prefoldin* genes in Arabidopsis (Hill and Hemmingsen, 2001). In this study, other three *prefoldins* (*At1g03760*, *At1g26660*, and *At1g49245*) were also found in this species. There are about 33,583, 39,454, and 42,577 genes in the *Arabidopsis*, maize and poplar genomes, respectively, which are 9.9, 29.2, and 39.4% larger than that of rice (30,534), respectively, implying a disproportion between the numbers of *prefoldin* genes and the sizes of predicted genomes. The *prefoldin* genes in plants encode highly hydrophilous polypeptides (from -0.003 to -0.988 in grand average hydrophobicities) with about from 16 to 612 amino acids and predicted pIs (from 4.21 to 10.2) (Supplementary Table S1). CELLO v2.5 server^10^ (Yu et al., 2004) was used to further predict the localization of the prefoldin proteins. The results showed that most of the candidate prefoldins were probably localized to the cytoplasm, suggesting that the prefoldin proteins participate in the cytoplasmic folding of tubulin and actin monomers (Zhao et al., 2005; Gu et al., 2008). Several plant prefoldins have also been localized in the nucleus (Supplementary Table S1), suggesting their functional relevance in DNA repair or integration, transcription and gene regulation (Millán-Zambrano and Chávez, 2014; Mirón-García et al., 2014).

**Table 1:** *Prefoldin* genes identified from 14 sequenced plant genomes.

Lineage	Organism	Genome size (Mb)^∗^	Number of predicted genes^∗^	Number of *prefoldin* genes
Algae	*Chlamydomonas reinhardtii*	120.41	14488	8
Moss	*Physcomitrella patens*	477.95	35936	9
Lycophytes	*Selaginella moellendorffii*	212.5	34782	7
Dicots	*Solanum lycopersicum*	781.51	27466	8
	*Vitis vinifera*	486.26	28268	9
	*Arabidopsis thaliana*	119.67	33583	9
	*Populus trichocarpa*	485.67	42577	13
	*Cucumis sativus*	244.82	21320	7
	*Medicago truncatula*	314.48	45000	6
	*Glycine max*	973.49	50202	24
Monocots	*Oryza sativa*	382.78	30534	10
	*Brachypodium distachyon*	272.06	26250	8
	*Zea mays*	2065.7	39454	13
	*Sorghum bicolor*	739.15	33081	9
**Total**				**140**

*^∗^The data come from www.ncbi.nlm.nih.gov/genome/*

### Phylogenetic Relationships, Gene Organization, and Motif Analysis

**FIGURE 1:**
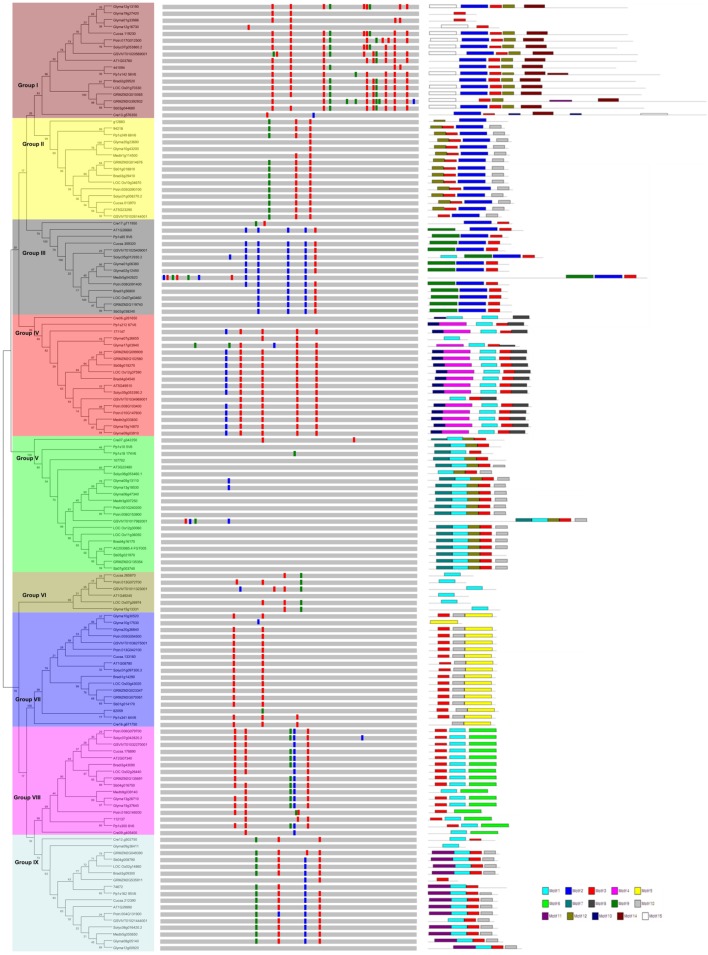
**Phylogenetic relationship, gene structure and motif composition of the *prefoldin* genes in plants**. The phylogenetic tree is constructed and classified into nine major groups (I–IX). The insertion positions of 0, 1, and 2 phase introns are marked with red, green, and blue lines, respectively. Different motifs of the prefoldin proteins are displayed by different colored boxes.

**FIGURE 2:**
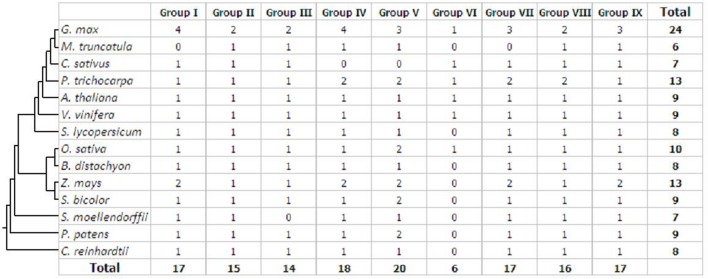
**Distribution of the *prefoldin* genes in different plant species and groups**.

To examine the phylogenetic relationships and evolutionary history of prefoldins in plants, I first constructed an unrooted tree and classified this gene family into nine groups (Group I-IX) (**Figure 1**) based on the observed topological structure of tree and sequence similarity. In addition, other methods, such as, maximum likelihood, PhyML methods, were also used to reconstruct the phylogenetic trees of prefoldin family, and very similar results were got as well as the tree topology of NJ method. Here, I employed the NJ tree for further analysis. This group classification was also supported by other evidences, such as intron-exon organization, and motif composition, which will be described below. Phylogenetic relationships of some eukaryotic prefoldin subunits have been illustrated (Hill and Hemmingsen, 2001). In which, six different clusters were generated with six prefoldin subunit sequences through phylogenetic analysis. In this study, I also found that all members of Groups II and IV had high homology with eukaryotic PFD5 and PFD3, respectively, which form α subunits of the prefoldin complex. While the members of Group V, Group VII, Group VIII, and Group IX represented four β subunits of PFD2, PFG4, PFD1 and PFD6, respectively. In addition to the six prefodlin subunit sequences, I also found and used three other *prefoldin* genes (At1g03760, At1g26660, and At1g49245) in *Arabidopsis* for BLAST searching. Because less conservation exists in prefoldin sequences, these sequences from 14 plant species were divided into nine groups (**Figure 1**). Each group contained 6–20 members. All terrestrial plants evolved from the primitive chlorophycean species in Viridiplantae (Misumi et al., 2008). Therefore, the origin of this gene may date back to the primitive algae. My search for *prefoldin* genes in *Chlamydomonas reinhardtii* yielded about eight members. This number remained relatively constant with the complexity of the genomes in plant evolution (**Figure 2**). The most obvious expansion of *prefoldin* gene family occurred in soybean, containing about 17% (24 paralogs) of the 140 identified *prefoldins*, which may be due to recent genome duplication occurring about 13 MYA (Schmutz et al., 2010).

In general, intron gain or loss generates structural complexity, which is a key evolutionary mechanism of most gene families (Cao, 2012; Chen and Cao, 2014; Cao et al., 2015). To further insight into the evolutionary relationships of the plant *prefoldin* genes, I investigated the exon-intron structure of each member and compared with each other. The position and distribution of introns of each *prefoldin* gene are shown in **Figure 1**. Each *prefoldin* gene contains 0–13 introns (**Figure 1**). Fifteen out of 20 members in Group V possessed a minimum of zero intron each, whereas *GRMZM2G392932* possessed a maximum of thirteen introns. Similar exon-intron structure occurred in most *prefoldin* genes of same group, implying conserved evolutionary relationship. For instance, most *prefoldin* gene in Group VII has two introns with same insertion position and phase distribution, whereas most members within Group IV contain five introns. The structural diversity in different groups suggests multiple origins of gene ancestry.

Next, to obtain more insights into the diversity of motif compositions in the 140 prefoldin proteins, I used the MEME motif search tool (Bailey et al., 2006) to search for the conserved motifs in these proteins, and found 15 conserved motifs (**Figure 1**). As described above, nine groups were classified among these *prefoldin* genes based on the phylogenetic analyses. Noticeably, I also found common motif compositions in most members of each group, implying functional similarities between them (**Figure 1**). Several groups (such as Groups I, IV, and V) possess five motifs, while Group VI only has one motif. Except for members in Group VI, motif 3 was shared by most other prefoldin proteins. I also found some additional distinct motifs in specific groups, such as, motif 2 restricted in Group I, II, and III; motifs 14 and 15 in Group I; motif 9 in Group III; and motif 13, 7, 6, and 11 in Group IV, V, VIII, and IX, respectively. Previous study has indicated that low conservation exists between prefoldin sequences (Hill and Hemmingsen, 2001). My motif composition analyses of the prefoldin proteins also confirm it. The diversity of the prefoldin sequences may increase the complexity of the binding substrate, thus greatly expand the functional scope of the prefoldin complex. Further investigation may be required to determine if any of these distinct motifs across diverse groups also have a unique functional role.

### Chromosomal Position and Duplication of *Prefoldin* Genes in Maize

**FIGURE 3:**
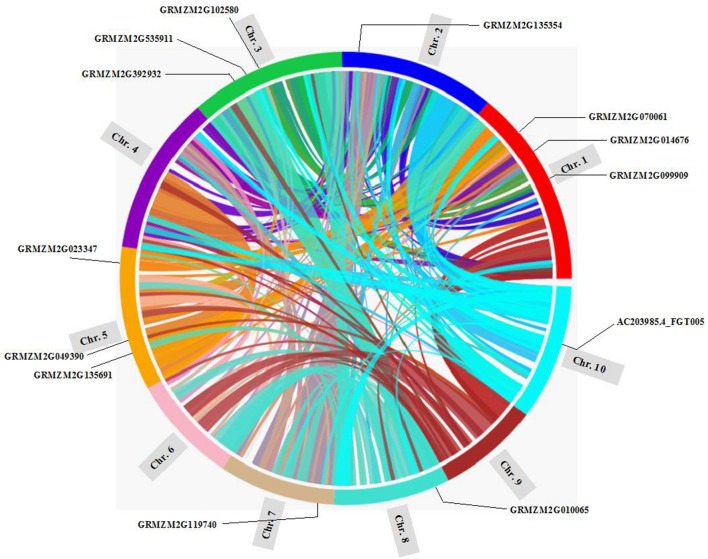
**Location of the *prefoldin* genes and segmental duplication regions on maize chromosomes**. The SyMAP database (Soderlund et al., 2011) was used to determine the segmental duplication regions.

Gene duplication, including segmental duplication, tandem duplication and retroposition, plays an important role in the evolution of organism (Chen Y et al., 2014; Cao and Li, 2015). To investigate the duplication mechanisms of *prefoldin* genes, I first examined their physical locations on chromosomes and found that the *prefoldin* genes were scattered in the maize genome (**Figure 3**). Further, all *prefoldin* genes were located in the duplicated segments of chromosomes in maize. Three of 6 pairs (*GRMZM2G023347/GRMZM2G070061*, *GRMZM2G099909/GRMZM2G102580*, and *GRMZM2G010065/GRMZM2G392932*) were retained in the maize duplication event (**Figure 3**). Next, I also used *K_s_* to estimate the evolutionary dates of duplicated *prefoldin* genes (**Table 2**). The results indicated that duplication events of maize 3/6 pairs occurred within the past 17.69–21.73 million years, consistent with the time of subsequent genome duplication events in maize (Gaut et al., 1996). In addition, the earliest segmental duplication event was also observed around 81.46 MYA in the *prefoldins* of maize (*GRMZM2G014676/GRMZM2G119740*) (Kellogg, 2001). Therefore, expansion of *prefoldin* genes in maize might have occurred due to the large-scale segmental duplication events in evolution. Segmental duplication contributes to the *prefoldin* family gene expansion in maize.

**Table 2:** Inference of duplication time of *prefoldin* paralogous pairs in maize.

Paralogous pairs	*K*_a_	*K*_s_	*K*_a/_*K*_s_	Data (million years ago)
*AC203985.4_FGT005/GRMZM2G135354*	0.01906	0.42986	0.04434	39.08
*GRMZM2G049390/GRMZM2G535911*	0.02581	0	–	0
*GRMZM2G023347/GRMZM2G070061*	0.00793	0.19994	0.03966	18.18
*GRMZM2G099909/GRMZM2G102580*	0.02172	0.23898	0.09089	21.73
*GRMZM2G010065/GRMZM2G392932*	0.09121	0.19455	0.46883	17.69
*GRMZM2G014676/GRMZM2G119740*	1.26364	0.89611	1.41014	81.46

### Functional Divergence Analyses of the Prefoldin Proteins

**FIGURE 4:**
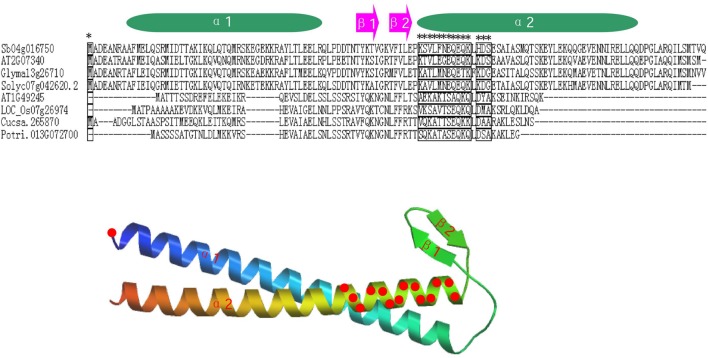
**Distribution of functional divergence sites of Group VI/VIII pairs**. Alignment results of some prefoldin proteins and the predicted tertiary structure are shown. Fifteen potential functional divergence sites are marked with a star in alignment results and marked with red circles on the predicted tertiary structure of the prefoldin protein.

To further study whether amino-acid substitutions cause adaptive functional diversification, the program DIVERGE (Gu, 1999, 2001) was used to estimate type-I functional divergence between different *prefoldin* groups. Thirty-six pairs of paralogous members were compared and estimated the evolutionary rate of each amino-acid sites. The results indicated that the coefficient of functional divergence (θ) values between 29 group pairs was less than 1 (**Table 3**), indicating significant site-specific alteration in selective constraints of most members of the prefoldin group pairs. Further, I also predicted a few critical residues associated with the functional divergence. For example, about 15 critical sites were predicted in Group I/II, I/VII, I/VIII, II/VII, V/VI, V/VII, VI/VIII, VII/VIII, and VII/IX pairs, while no site was predicted in the Group II/IX, IV/IX, and V/IX pairs. An example of detailed distribution of the functional divergence sites of Group VI/VIII pairs was shown in **Figure 4**. Further analyses indicated that one divergence site is located in the first residue. Other fourteen predicted sites are located in the N-terminal of α-helice 2 (α2). As we know, prefoldin can form a heterohexameric jellyfish like structure with coiled-coil tentacles (Siegert et al., 2000). These tentacles are involved in substrate binding. In this study, I found that some amino-acid sites on α-helice 2 (tentacle) present different sequence composition, implying an increase in substrate specificity for target proteins. Higher theta values (θ) exit in Group I/VI, I/VII, I/VIII, V/VI, V/VII, VII/VIII, and VII/IX pairs (**Table 3**), suggesting a higher evolutionary rate between them. Thus, due to the different evolutionary rates, the *prefoldin* genes diverge functionally from each other. The amino-acid mutations promoted the functional evolution and divergence of *prefoldin* genes, as an adaptation of the species to the changing environment (Cao et al., 2011).

**Table 3:** Functional divergence estimated in *prefoldin* paralogs.

Groups comparison	θ^1^	*SE*^2^	LRT^3^	N(0.5)^4^	*N*(0.8)^4^
I/II	0.826543	0.239298	17.32376	15	15
I/III	0.803522	0.190311	12.43868	13	4
I/IV	0.646644	0.285272	8.070123	15	7
I/V	0.027228	0.201279	1.794084	1	1
I/VI	1.105756	0.246338	13.44324	15	14
I/VII	1.689677	0.300763	31.56156	15	15
I/VIII	1.053435	0.198181	25.42036	15	15
I/IX	0.014082	0.230382	1.732052	1	1
II/III	0.765629	0.156564	12.61142	6	3
II/IV	0.960633	0.189864	18.39889	15	8
II/V	0.332706	0.276028	3.636844	6	2
II/VI	0.404784	0.300171	0.682597	1	0
II/VII	0.977057	0.268244	13.87541	15	15
II/VIII	0.811230	0.216721	13.90022	15	6
II/IX	-0.278640	0.410256	0.078849	0	0
III/IV	0.514812	0.177764	6.860248	4	1
III/V	0.507173	0.345447	0.811551	1	0
III/VI	0.976963	0.304516	8.12796	15	13
III/VII	0.187283	0.378168	1.031081	3	0
III/VIII	0.996719	0.203875	19.93697	15	14
III/IX	0.199838	0.371056	0.945485	1	0
IV/V	0.483363	0.269882	1.506023	1	1
IV/VI	0.563231	0.320949	6.915311	15	12
IV/VII	0.636617	0.225039	13.53771	15	11
IV/VIII	0.254421	0.133029	4.686944	2	1
IV/IX	-0.389670	-0.851500	0.209424	0	0
V/VI	1.083988	0.393359	7.594005	15	15
V/VII	1.395175	0.280780	12.16217	15	15
V/VIII	0.844957	0.220248	10.71504	14	4
V/IX	-0.619080	-0.380780	2.643254	0	0
VI/VII	0.515722	0.419894	1.993136	13	2
VI/VIII	0.932783	0.312437	10.2438	15	15
VI/IX	0.359807	1.001866	0.075892	0	0
VII/VIII	1.021701	0.258359	12.72445	15	15
VII/IX	1.647830	0.628639	6.871029	15	15
VIII/IX	-0.048070	0.311906	0.876434	1	0

*^1^θ: the coefficient of functional divergence. ^2^SE: standard error. ^3^LRT: likelihood ratio test. ^4^N(0.5) and N(0.8): the numbers of divergent residues when the cut-off value is 0.5 and 0.8, respectively*.

### Functional Network Analysis of the Maize Prefoldins

**FIGURE 5:**
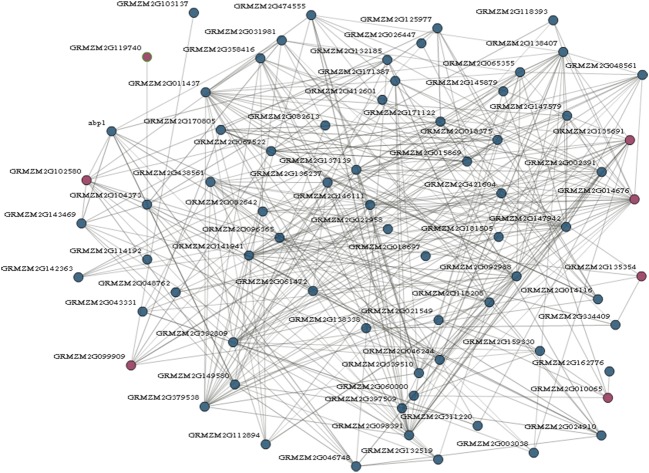
**Prefoldin genes interaction network**. Seven prefoldin genes are mapped to the co-expression database and reveal a total of 78 unique genes that showed 347 interactions in maize.

Genes involved in the same biological process are often coordinately expressed, and thus their co-expression information will be as a factor to understand the biological process (Eisen et al., 1998). To further explore which genes are possibly interacted by maize prefoldin, I performed a coexpression analyses with the COB^11^ (Schaefer et al., 2014). I found that 7 of 13 maize prefoldins were co-expressed in this network, resulting in 347 interactions exhibited by 78 genes (**Figure 5**). Among the 347 interactors identified, 23 and 17 genes were coexpressed with *GRMZM2G014676* and *GRMZM2G135691*, respectively. *GRMZM1G014676* is an orthologous gene of *Arabidopsis PFD5* (*At5g23290*), while *GRMZM2G135691* is an orthologous gene of *Arabidopsis PFD1* (*At2g07340*) (**Figure 1**). Moreover, PFD5 plays an essential role in *Arabidopsis* tolerance to salt stress (Rodríguez-Milla and Salinas, 2009). They may also have similar biology functions. The network analysis of maize prefoldins has a reference value for the functional research of *prefoldin* genes. Some of these interactors include carrier proteins, such as equilibrative nucleoside transporter (ENT), mitochondrial phosphate carrier (MPC), acyl carrier protein (ACP), and so on. ENT (GRMZM2G002391) -mediated nucleosides transport across plasma membrane influenced Arabidopsis growth and pollen germination (Bernard et al., 2011). MPC (GRMZM2G118208) is a mitochondrial solution carrier protein, which delivers phosphate across the inner mitochondrial membrane and function in plant development and resistance (Haferkamp and Schmitz-Esser, 2012). As a small acidic protein with conserved Asp-Ser-Leu (DSL) motif, ACP (GRMZM2G149580) functions in root nodule symbiosis in soybean (Wang et al., 2014). I also found that another interactor, an auxin binding protein (ABP1), is required for binding to auxin at low concentration and involved in the embryogenesis, auxin signaling and cell division (Chen X et al., 2014; Xu et al., 2014). In addition, an *Arabidopsis* cyclin-dependent kinase (CDK) G2 regulated the salinity stress response and was associated with the control of flowering time (Ma et al., 2015). Coexpression analyses reflect the correlation of the expression profiles of different genes, and are suggestive in tracing the genes in the same biological process or pathway. My results indicated that some proteins possessed carrying, binding, and kinase function were usually coexpressed with maize prefoldins (**Figure 5**). The *prefoldin* genes may be involved in some related molecular processes by interacting with these interactors. Thus, whether these interactors could function with prefoldin need further experimental verification. The approaches and results open a new way to explore the molecular mechanism of prefoldin dynamics. Also, this interaction can also help to study the key regulatory steps in these processes or pathways. It will be advantageous to screen candidate genes for further functional researches.

### Expression Patterns and Promoter Sequence Analysis of the Maize *Prefoldin* Genes

**FIGURE 6:**
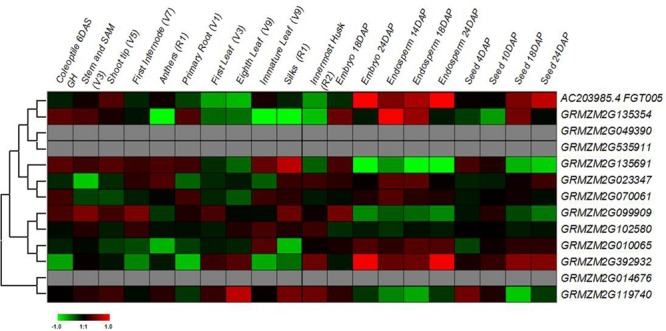
**Expression profiles of the maize *prefoldin* genes in 20 different developmental stages**.

The gene expression patterns may provide important clues to their functions (Cao and Shi, 2012). Here, I first tested spatial and temporal specific expression patterns of maize *prefoldin* genes in 20 tissues using microarray data. I detected 10 probes of the maize *prefoldin* transcripts, and other three transcripts (*GRMZM2G049390*, *GRMZM2G535911*, and *GRMZM2G014676*) with no detectable probes were not further analyzed in this study. The results showed that the expression abundance of the *prefoldin* genes detected presented great variability in maize (**Figure 6**). Some *prefoldins* (*AC203985.4_FGT005*, *GRMZM2G135354*, and *GRMZM2G392932*) expressed exclusively in developing embryo and endosperm may improve seed yield and quality. Notably, due to the great expression in maize silk, *GRMZM2G135691* may play a key role in silk development.

**FIGURE 7:**
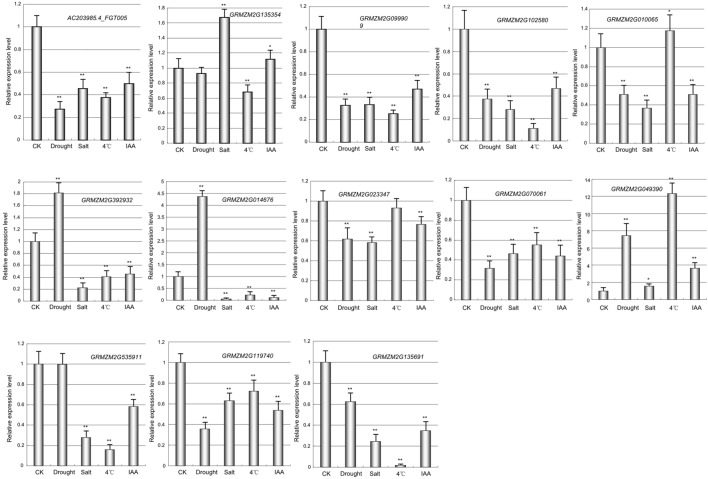
**Expression profiles of the maize *prefoldin* genes under abiotic stresses and IAA**. Experiments were conducted in triplicate. Significance was tested relative to each CK using *t*-test. Significance of ^∗^*P* < 0.05 and ^∗∗^*P* < 0.01. Error bar: standard deviation.

Next, I also investigated the responses of maize *prefoldin* genes under drought, low temperature and salt stresses using 10-day-old seedlings. Quantitative RT-PCR was used to analyze the expression profiles of 13 *prefoldin* genes in maize under various stresses (**Figure 7**). The expression of *GRMZM2G135354* and *GRMZM2G049390* was significantly upregulated during drought, whereas other *prefoldin* genes were downregulated. The expression of over 84 percent of maize *prefoldins* was significantly repressed under salt, whereas *GRMZM2G135354* and *GRMZM2G049390* were strongly induced, suggesting that the *prefoldin* genes might present different responses to salinity or drought stress. Similar results also occurred with low-temperature. Under low temperature stress, most *prefoldin* genes were significantly downregulated (**Figure 7**). For instance, the expression level of *GRMZM2G135691* decreased about 50 times. However, the expression of some *prefoldin* genes (*GRMZM2G010065*, and *GRMZM2G049390*) was induced. Previous study has shown that osmotic stress caused by drought and salinity can change microtubule orientation, and then regulates primary root elongation in *Arabidopsis* (Liu et al., 2014). Moreover, low temperature also leads to the depolymerization of microtubule (Bartolo and Carter, 1991). My expression profiles of maize *prefoldin* genes under these stresses indicated that most *prefoldin* members were significantly repressed. Therefore, it can be inferred that under the drought, salinity, or low temperature stress, the decreased expression of *prefoldin* genes may inhibit or change the development of microtubules. Next, the auxin response of these maize *prefoldin* genes was also investigated. Among the 13 *prefoldin* genes detected, three members (*GRMZM2G392932*, *GRMZM2G014676*, and *GRMZM2G049390*) were upregulated during IAA treatment, suggesting important roles in regulating IAA response. Nevertheless, the expression of *GRMZM2G135354* and *GRMZM2G535911* was unchanged after IAA treatment, whereas others were steadily decreased during the treatment (**Figure 7**).

**FIGURE 8:**
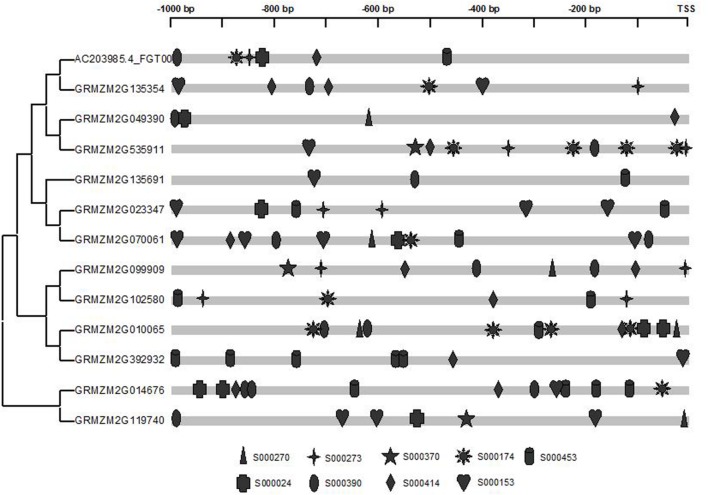
**Promoter analyses of the maize *prefoldin* genes**. *Cis*-regulated elements responsive to abiotic stresses and phytohormones are marked differently.

To further explore the potential regulatory mechanism of maize *prefoldin* genes in phytohormone and abiotic stress stimuli, I used the PLACE web server^12^ (Higo et al., 1999) to identify their putative *cis*-elements. Here, 1,000-bp promoter regions of the maize *prefoldin* genes were analyzed. A few phytohormones and abiotic response regulatory elements were found. They included auxin response factor (ARF)-binding site (S000270); tissue-specific expression and auxin-inducible (S000273); auxin-inducible (S000370); IAA/SA-inducible (S000024); SA-inducible (S000390); drought-inducible (S000414 and S000174); cold/drought-inducible (S000153); and salt-inducible (S000453) response regulatory elements. I also found that all maize *prefoldin* genes contained multiple regulatory elements in their promoter regions (**Figure 8**), suggesting that these phytohormones and abiotic stresses regulated the expression of maize *prefoldin* genes. Comparing the distribution of nine *cis*-elements in their promoter sequences, I found variation between all sister pairs of maize *prefoldin* genes, implying divergent expression profiles in the duplicated genes. It may be the reason of subfunctionalization or neofunctionalization (Prince and Pickett, 2002).

## Summary

I performed a comparative analysis of the plant *prefoldin* gene family in this study, and found that the prefoldin family could be divided into nine groups by phylogenetic analyses. Gene organization and motif compositions of the prefoldin members were highly conserved in each group, implying their functional conservation. Segmental duplication contributed to maize *prefoldin* family gene expansion. A few critical residues associated with the functional divergence were predicted. Functional network analyses identified some co-expressed genes, which usually have binding, carrying and kinase activity. Furthermore, the differential expression profiles of the maize *prefoldin* genes suggested functional divergence during development and IAA and some abiotic stress treatments. Some *cis*-elements responsive to abiotic stress and phytohormone were also found in the upstream sequence of the maize *prefoldin* genes. These data will provide useful insights for further functional investigation of this gene family in the future.

## Author Contributions

JC designed and carried out the experiments and wrote the manuscript. The author read and approved the manuscript.

## Conflict of Interest Statement

The author declares that the research was conducted in the absence of any commercial or financial relationships that could be construed as a potential conflict of interest. The reviewer AB and the handling Editor declared a shared affiliation, and the handling Editor states that the process nevertheless met the standards of a fair and objective review.

## Footnotes

1 http://www.phytozome.net

2 http://web.expasy.org/protparam

3 http://cello.life.nctu.edu.tw

4 http://www.maizesequence.org

5 http://www.phytozome.net

6 http://meme.sdsc.edu

7 http://csbio.cs.umn.edu/cob

8 http://www.dna.affrc.go.jp/PLACE/signalscan.html

9 http://www.phytozome.net

10 http://cello.life.nctu.edu.tw

11 http://csbio.cs.umn.edu/cob

12 http://www.dna.affrc.go.jp/PLACE/
