# Supplementary material for: Analysis of the Prefoldin Gene Family in 14 Plant Species
Source: Front Plant Sci. 2016 Mar 15;7:317. doi: 10.3389/fpls.2016.00317 (PMC4792155; doi:10.3389/fpls.2016.00317)
Supplement: Supplementary file 2 [file Data_Sheet_2.DOC]

**Supplementary Table 2** Primers used in this study.

| **Primer names** | **Primer sequence (5`-3`)** |
| --- | --- |
| AC203985.4_FGT005-F | CAAGGAGGGCCTGGAAGAG |
| AC203985.4_FGT005-R | GCTCAAACTCGGTTATCTCCTTCT |
| GRMZM2G135354-F | AAAGAAGCCATAAACGAGCAAGTAG |
| GRMZM2G135354-R | TCCAGCTCTGTGATCTTGGTATAGA |
| GRMZM2G099909-F | GAAGTCCTTGTTGCTGATCTTCAG |
| GRMZM2G099909-R | TGAATACCCGAGCAATTGTAACC |
| GRMZM2G102580-F | AAGTCCTTGTTGCTGATCTCCATT |
| GRMZM2G102580-R | TGAATACCCGAGCAATTGTAACC |
| GRMZM2G010065-F | AGCCATCATCCCATACATCTCAT |
| GRMZM2G010065-R | TTTAGTGTGAACTCGGACATCAGAA |
| GRMZM2G392932-F | TCCTACCTTGCTAGTGGGTTCAA |
| GRMZM2G392932-R | TCGCCCACTCGATATTATCTTTC |
| GRMZM2G014676-F | CTGGACGACGCGGAGAAG |
| GRMZM2G014676-R | CCCCTGAGCCATGGTTTTC |
| GRMZM2G023347-F | GAGGCTAGAGGAGGAGAAGGAATC |
| GRMZM2G023347-R | TCCTTGAACTTCCCGTACAGTATCT |
| GRMZM2G070061-F | GGGAGGACCAGCAGAACATC |
| GRMZM2G070061-R | TCCTTCGCGAGCTTGATCTC |
| GRMZM2G049390-F | ACCGGTGCTGGTCAAGCA |
| GRMZM2G049390-R | CTTTGACAGCTCTGCGGAGAT |
| GRMZM2G535911-F | GGACCGGTGCTGGTCAAG |
| GRMZM2G535911-R | CCGATCCATCCGCTTCAG |
| GRMZM2G119740-F | ATTGAAAATTTGGAGAGGAATGGT |
| GRMZM2G119740-R | TCTGCTTGCATGTACACCTCTGA |
| GRMZM2G135691-F | GCTGCAGTCCCGGATGAT |
| GRMZM2G135691-R | GCGAAGTTCCTCCAAAGTAAGG |
| Actin 1-F | CGAATGCCCAGCAATGTATGT |
| Actin 1-R | GCTCACACCATCACCGGAAT |
